# Characterization of Multidrug-Resistant *Salmonella enterica* Serovars Indiana and Enteritidis from Chickens in Eastern China

**DOI:** 10.1371/journal.pone.0096050

**Published:** 2014-05-02

**Authors:** Yan Lu, Hongyu Zhao, Jian Sun, Yuqi Liu, Xuping Zhou, Ross C. Beier, Guojuan Wu, Xiaolin Hou

**Affiliations:** 1 College of Animal Science and Technology, Beijing University of Agriculture, Beijing, China; 2 National Institute of Biological Sciences, Beijing, China; 3 Department of Animal Husbandry and Veterinary Medicine, Beijing Vocational College Agriculture, Beijing, China; 4 USDA, Agricultural Research Service, Southern Plains Agricultural Research Center, Food and Feed Safety Research Unit, College Station, Texas, United States of America; Rockefeller University, United States of America

## Abstract

A total of 310 *Salmonella* isolates were isolated from 6 broiler farms in Eastern China, serotyped according to the Kauffmann-White classification. All isolates were examined for susceptibility to 17 commonly used antimicrobial agents, representative isolates were examined for resistance genes and class I integrons using PCR technology. Clonality was determined by pulsed-field gel electrophoresis (PFGE). There were two serotypes detected in the 310 *Salmonella* strains, which included 133 *Salmonella enterica* serovar Indiana isolates and 177 *Salmonella enterica* serovar Enteritidis isolates. Antimicrobial sensitivity results showed that the isolates were generally resistant to sulfamethoxazole, ampicillin, tetracycline, doxycycline and trimethoprim, and 95% of the isolates sensitive to amikacin and polymyxin. Among all *Salmonella enterica* serovar Indiana isolates, 108 (81.2%) possessed the *bla*_TEM_, *flo*R, *tet*A, *str*A and *aac (6')-Ib-cr* resistance genes. The detected carriage rate of class 1 integrons was 66.5% (206/310), with 6 strains carrying gene integron cassette *dfr*17-*aad*A5. The increasing frequency of multidrug resistance rate in *Salmonella* was associated with increasing prevalence of *int*1 genes (r_s_ = 0.938, *P* = 0.00039). The *int*1, *bla*_TEM_, *flo*R, *tet*A, *str*A and *aac (6')-Ib-cr* positive *Salmonella enterica* serovar Indiana isolates showed five major patterns as determined by PFGE. Most isolates exhibited the common PFGE patterns found from the chicken farms, suggesting that many multidrug-resistant isolates of *Salmonella enterica* serovar Indiana prevailed in these sources. Some isolates with similar antimicrobial resistance patterns represented a variety of *Salmonella enterica* serovar Indiana genotypes, and were derived from a different clone.

## Introduction

*Salmonella* is a leading cause of microbial food poisoning cases and salmonellosis is one of the most important bacterial diseases in chicken farms, leading to large numbers of chicken death and significant loss to the poultry industry [1]. The widespread use of antimicrobial agents in food-animal production has contributed to the decreased susceptibility of *Salmonella* to antibiotics, which can be transmitted to humans through food of animal origin [2]. In recent years, the rapid emergence of multidrug resistance bacteria caused by the wide application of antibiotics in clinical practices has caused great difficulties in medical treatment [3]. The integron gene cassette system is one of the main reasons for the rapid development of multidrug resistance in *Salmonella* [4]. The same gene cassette found in different genotype strains indicates that it can spread in clinical strains through horizontal transfer. Moreover, integrons can propagate vertically [5]–[6]. The existence and flexible transmission of integrons was proven suitable for the spread of drug resistant genes and the acceleration of multidrug resistance [7]. In the current study, the drug sensitivity of *Salmonella* strains isolated in chicken farms from Eastern China in 2009 was tested, and the prevalent class 1 integrons and integron-carrying gene cassette in connection with resistance in the strains was studied. We assessed the relationship between multidrug resistance of *Salmonella* and class 1 integrons by statistical analyses.

## Materials and Methods

### *Salmonella* isolation, identification and serotyping

A total of 1024 strains were collected in 2008–2009 from 6 broiler farms in 3 regions of Eastern China. Fecal samples were taken in cloaca with disposable sterile swabs from two chicken farms in Lin Yi (280 samples), one chicken farm in Zou Ping (190 samples) and three chicken farms in Yan Tai (554 samples). Dr. Yuqing Liu (Institute of Animal Science and Veterinary Medicine Shandong Academy of Agricultural Sciences, China) responsible for contact takes the strains. The strains were collected according to acquisition guidelines. The field studies did not involve endangered or protected species.

After the samples incubated in sterile Selenite Cystine Broth (SC) at 37°C for 24 h, all of the samples incubated were grown on chromogenic medium for *Salmonella* (CHROM agar, Paris, France) at 37°C for 24–48 h. Only one colony per plate was picked for further study, and purple-colored colonies on the culture plates were regarded as presumptive *Salmonella* colonies. Suspected colonies were isolated and grown on nutrient agar (Dibco), and then identified by transferring to tubes with triple sugar iron agar, indole-lysin motility semisolid agar, Voges Proskauer semisolid media, urease test broth, and Simmons citrate agar. The isolates examined with these media were simultaneously investigated for presence of the *inv*A gene with the polymerase chain reaction (PCR).

*Salmonella* isolates were serotyped based on slide agglutination for O and H antigens according to Kauffmann-White [8], and using the same antisera produced at the National Institute of Biological Sciences in Beijing.

Antimicrobial sensitivity

Minimum inhibitory concentrations (MICs) of the *Salmonella* isolates were determined by broth micro dilution according to the guidelines of the Clinical and Laboratory Standards Institute (CLSI) [9]–[10]. *Escherichia coli* ATCC 25922 was used as the quality control strain. Seventeen antimicrobials were tested (MIC break point Sensitivity/Resistance µg/mL): namely, ampicillin (≤8/≥32), amoxicillin-clavulanic acid (≤4/≥16), cefazolin (≤8/≥32), ceftiofur (≤2/≥8), tetracycline (≤4/≥16), doxycycline (≤4/≥16), chloramphenicol (≤8/≥32), florfenicol (≤4/≥16), kanamycin (≤16/≥64), gentamicin (≤4/≥16), sulfisoxazole (≤256/≥512), trimethoprim (≤38/≥76), enrofloxacin (≤0.25/≥2), norfloxacin (≤4/≥16), ciprofloxacin (≤0.06/≥2), amikacin (≤16/≥64) and polymyxin (≤2/≥4).

### PCR amplification and DNA sequencing of resistance genes and integrase genes

According to the MIC data, multidrug-resistant genes of 133 *Salmonella enterica* serovar Indiana and 177 *Salmonella enterica* serovar Enteritidis were analyzed by PCR. Antimicrobial resistance genes were detected for ampicillin-resistant isolates (*bla*_TEM_ and *bla*_PSE_); chloramphenicol/florfenicol-resistant isolates (*cat*A1, *cat*A2, *cat*A3, *cml*A and *flo*R); tetracycline-resistant isolates (*tet*A, *tet*B, *tet*C, *tet*D and *tet*G), streptomycin-resistant isolates (*aad*A, *str*A and *str*B), fluoroquinolone-resistant isolates (*qnr*A, *qnr*B, *qnr*S, *aac(6′)-Ib-cr* and *qep*A), and integrase genes (*int*1, *int*2 and *int*3) [11]. The DNA sequences obtained were compared with those in GenBank using the BLAST program.

### Pulsed field gel electrophoresis (PFGE)

Chromosomal DNA of 104 *Salmonella enterica* serovar Indiana isolates carrying the *int*1, *bla*_TEM_, *flo*R, *tet*A, *str*A, and *aac(6’)-Ib-cr* genes were digested with the restriction enzyme *Xba*I and then subjected to PFGE analysis according to the Pulse Net Standardized Laboratory Protocol (U.S. Centers for Disease Control and Prevention, Atlanta, GA) using the CHEF MAPPER System (Bio-Rad Laboratories, Hercules, CA). The gels were run at 6.0 V/cm with an initial and final switch time of 2.16 s and 54.17 s at an angle of 120 degrees and 14°C for 18 h. The *Salmonellaser*. Braenderup H9812 standard served as size markers. Cluster analysis of pulsotypes was carried out using Dice's coefficient in UPGMA with InfoQuest FP Software/Version 4.5 (Bio-Rad). [12]

### Statistical analysis

The relation between multidrug resistance rate and prevalence of *int*1 genes was assessed by calculating simple linear regression and the corresponding *P* value. All statistical analyses were done using SPSS software (SPSS, Chicago, IL).

## Results

### Strain identification and serotyping

A total of two serotypes were identified among 310 strains of *Salmonella*, accounting for 133 strains of *Salmonella enterica* serovar Indiana and 177 strains of *Salmonella enterica* serovar Enteritidis. No other serotypes were identified in all *Salmonella* isolates.

### Antimicrobial sensitivity and multidrug resistance

As shown in Table 1, the resistance rates of the *Salmonella enterica* serovar Indiana isolates were 100% to sulfamethoxazole; above 90% to ampicillin, florfenicol, tetracycline, doxycycline, kanamycin, gentamicin and trimethoprim; above 80% to amoxicillin/clavulanic acid, cefazolin, ceftiofurand chloramphenicol; over 60% to enrofloxacin, norfloxacin and ciprofloxacin; and about 5% to amikacin and polymyxin E. Antimicrobial sensitivity showed that the *Salmonella enterica* serovar Indiana strains were multidrug resistant and only sensitive to amikacin and polymyxin. The isolates of *Salmonella enterica* serovar Enteritidis were resistant to ampicillin, tetracycline, doxycycline and trimethoprim above 60%, and were sensitive to all other tested drugs.

**Table 1:** Number and antimicrobial resistance profiles of resistant *Salmonella* strains within each serogroup.

Sources (no. of isolates)	Serovar (no. of isolates)	Percentage of resistance (no. of isolates)																
		AMP	AMC	CEF	XNL	CHL	FFN	TET	DOX	KAN	GEN	AMI	SUL	TMP	ENR	NOR	CIP	POL
Lin Yi (119)	Indiana (5)	100 (5)	100 (5)	60 (3)	60 (3)	100 (5)	60 (3)	100 (5)	100 (5)	60 (3)	60 (3)	0	100 (5)	100 (5)	60 (3)	80 (4)	40 (2)	0
	Enteritidis (114)	78.1 (89)	42.1 (48)	5.3 (6)	6.1 (7)	5.3 (6)	0.9 (1)	73.7 (84)	73.7 (84)	9.6 (11)	1.8 (2)	0	100(114)	74.6 (85)	0	2.7 (3)	0.9 (1)	0.9 (1)
Zou Ping (51)	Indiana (28)	96.4 (27)	82.1 (23)	92.9 (26)	85.7 (24)	75 (21)	96.4 (27)	89.3 (25)	92.9 (26)	82.1 (23)	85.7 (24)	17.9(5)	100 (28)	96.4 (27)	53.6(15)	71.4 (20)	53.6(15)	7.1 (2)
	Enteritidis (23)	69.6 (16)	17.4 (4)	17.4 (4)	13.0 (3)	4.3 (1)	0	52.2 (12)	43.5 (10)	4.3 (1)	4.3(1)	4.3(1)	100 (23)	60.9 (14)	0	4.3(1)	13.0 (3)	0
Yan Tai (140)	Indiana (100)	98 (98)	89 (89)	88 (88)	87 (87)	87 (87)	91 (91)	100 (100)	100 (100)	94 (94)	96 (96)	1 (1)	100(100)	100 (100)	69 (69)	81 (81)	62 (62)	5 (5)
	Enteritidis (40)	62.5 (25)	17.5 (7)	5 (2)	5 (2)	0	0	55 (22)	47.5 (19)	2.5(1)	12.5(5)	2.5(1)	100 (40)	30 (12)	0	2.5 (1)	2.5 (1)	12.5(5)
Total (310)	Indiana (133)	97.7 (130)	887.9 (117)	87.9 (117)	85.7(114)	84.9(113)	90.9(121)	97.7(130)	98.5(131)	90.2(120)	92.5(123)	4.5(6)	100(133)	99.2(132)	65.4(87)	78.9(105)	59.4(79)	5.3 (7)
	Enteritidis (177)	73 (130)	33.3 (59)	6.8 (12)	6.8 (12)	3.9 (7)	0.6 (1)	66.7(118)	63.8(113)	7.3(13)	4.5(8)	1.1(2)	100(177)	62.7(111)	0	2.8(5)	2.8 (5)	3.4 (6)

AMP, ampicillin; AMC, amoxicillin/clavulanic acid; CEF, cefalotin; XNL, ceftiofur; CHL, chloramphenicol; FFN, florfenicol; TET, tetracycline; DOX, doxycycline; KAN, kanamycin; GEN, gentamicin; AMI, amikacin; SUL, sulfamethoxazole; TMP, trimethoprim; ENR, enrofloxacin; NOR, norfloxacin; CIP, ciprofloxacin; POL, polymyxin.

**Figure 1:**
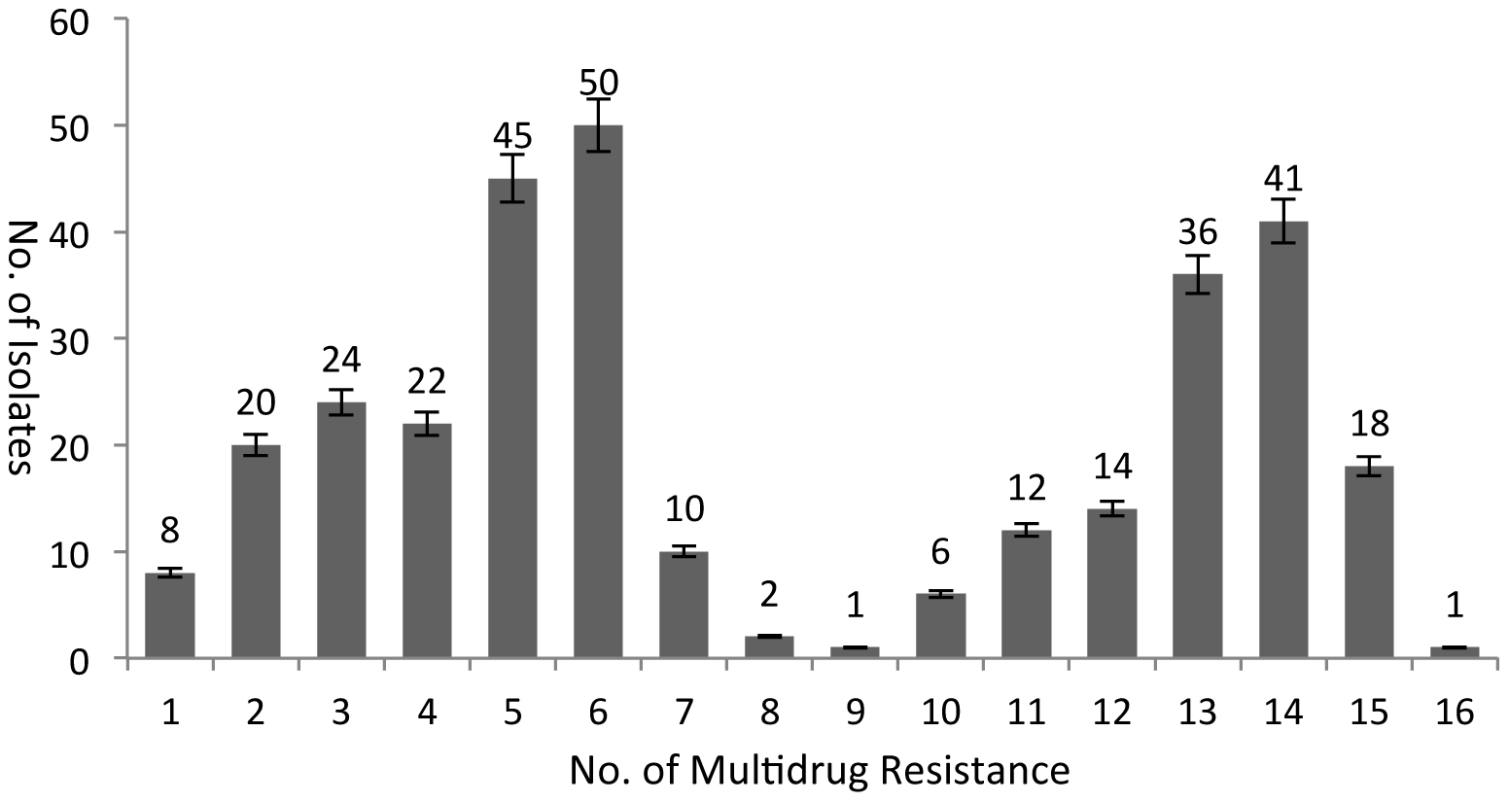
No. of multidrug resistance to 17 drugs in 310 Salmonella isolates. The 310 strains of *Salmonella* were strongly resistant to 17 antibiotics generally used in clinical practice. The most common *Salmonella* strains were resistant to 5 and 6 drugs of 44 and 50 strains. The next most common was *Salmonella* with 13 and 14 drug resistance of 36 and 41 strains.

The 310 strains of *Salmonella* were strongly resistant to 17 antibiotics generally used in clinical practice (Figure 1). The most common *Salmonella* strains were resistant to 5 and 6 drugs of 44 and 50 strains, respectively, accounting for 30.6% of the total, with most represented by *Salmonella enterica* serovar Enteritidis. The next most common was *Salmonella* with 13 and 14 drug resistance of 36 and 41 strains, respectively, accounting for 24.8% of the total, with most represented by *Salmonella enterica* serovar Indiana, whose drug resistance of the two serotypes showed double peaks. A total of 74 (23.8%) *Salmonella* strains were below 4 drug resistance, 45 (14.5%) *Salmonella* strains had 7 to 12 drug resistance, 18 (5.8%) *Salmonella* strains had 15 drug resistance, and one *Salmonella* strain had 16 drug resistance. *Salmonella* with 15 and 16 drug resistances were *Salmonella enterica* serovar Indiana. No strain of *Salmonella* showed zero drug resistance. The above results showed that *Salmonella* with above 10 drug resistance were mainly *Salmonella enterica* serovar Indiana. A total of 282 (90.9%) *Salmonella* strains were resistant to more than 3 kind of drug, demonstrating its multidrug resistance.

### PCR amplification and DNA sequencing resistance genes

As shown in Table 2, *bla*_TEM_, *cat*A1, *flo*R, *tet*A, *str*A and *aac(6′)-Ib-cr* were the most prevalent resistance genes and were present in *Salmonella enterica* serovar Indiana isolates from all chicken farm sources. The *bla*_TEM_ gene was prevalent in *Salmonella enterica* serovar Indiana (78.2%) and *Salmonella enterica* serovar Enteritidis (62.4%) isolates from the three sources. The *flo*R, *tet*A and *str*A genes were highly prevalent in *Salmonella enterica* serovar Indiana isolates, and were detected in 92% of the isolates from the three chicken farms. The *aac (6′)-Ib-cr* gene was highly prevalent in *Salmonella enterica* serovar Indiana isolates, and was present in 95 (95%) of the 100 isolates from Yan Tai chicken farm, 25 (89.3%) of the 28 isolates from ZouPing chicken farm, and 3 (60%) of the 5 isolates from Lin Yi chicken farm. Among all *Salmonella enterica* serovar Indiana isolates, 108 (81.2%) possessed the *bla*_TEM_, *flo*R, *tet*A, *str*A and *aac(6')-Ib-cr* resistance genes, which were the most frequently observed combination in this study.

**Table 2:** Distribution of antimicrobial resistance genes and integrase genes among *Salmonella*.

Sourcce (no. of isolates)	Serovar (no. of isolates)	Percentage (no.) of isolates carrying resistance gens and integrase gens								
		*bla*_TEM_	*cat*A1	*flo*R	*clm*A	*tet*A	*str*A	*aac(6′)-Ib-cr*	*int*1	Multidrug resistance rate (no. of isolates)
Lin Yi (119)	Indiana (5)	80 (4)	40 (2)	60 (3)	0	60(3)	60(3)	60(3)	80 (4)	100 (5)
	Enteritidis (114)	69.6 (80)	4.3 (5)	5.2 (6)	1.7(2)	3.5(4)	2.6(3)	0	57 (65)	88.6 (101)
Zou Ping (51)	Indiana (28)	89.3 (25)	89.3 (25)	100 (28)	0	96.4(27)	96.4(27)	89.3(25)	96.4 (27)	100 (28)
	Enteritidis (23)	56.5 (13)	8.7 (2)	4.4 (1)	0	0	0	0	43.5 (10)	82.6 (19)
Yan Tai (140)	Indiana (100)	75 (75)	77 (77)	97(97)	7(7)	94(94)	93(93)	95(95)	85 (85)	96 (96)
	Enteritidis (40)	45 (18)	0	0	2.5(1)	0	0	0	37.5 (15)	82.5 (33)
Total (310)	Indiana (133)	78.2 (104)	78.2 (104)	96.2(128)	5.3(7)	93.2(124)	92.5(123)	92.5(123)	87.2 (116)	97 (129)
	Enteritidis (177)	62.4 (111)	3.9 (7)	3.9(7)	1.7(3)	2.2(4)	1.7(3)	0	50.8 (90)	86.4 (153)

### Prevalence of class I integrons

Among the 310 *Salmonella* strains, 206 were amplified to *int*1 fragments of about 856 bp with a positive rate of 66.5%. The *int*1 positive rate was 87.2% (116) for the 133 *Salmonella enterica* serovar Indiana isolates and 50.8% (90) for the 177 *Salmonella enterica* serovar Enteritidis isolates. The increasing frequency of multidrug resistance rate in *Salmonella* was associated with increasing prevalence of *int*1 genes (r^2^ = 0.938, *P* = 0.00039). One 1515 bp amplicon was obtained from the variable region of the class 1 integrons by PCR. Sequencing and nucleotide sequence analysis of gene cassettes deposited in GenBank showed that this amplicon contained *dfr*17 and *aad*A5 with a detection rate of 2.91% (6/206). The drug resistant gene cassette *dfr*17-*aad*A5 was correlated with drug resistance to trimethoprim and aminoglycosides, respectively.

### Pulsed-field gel electrophoresis

**Figure 2:**
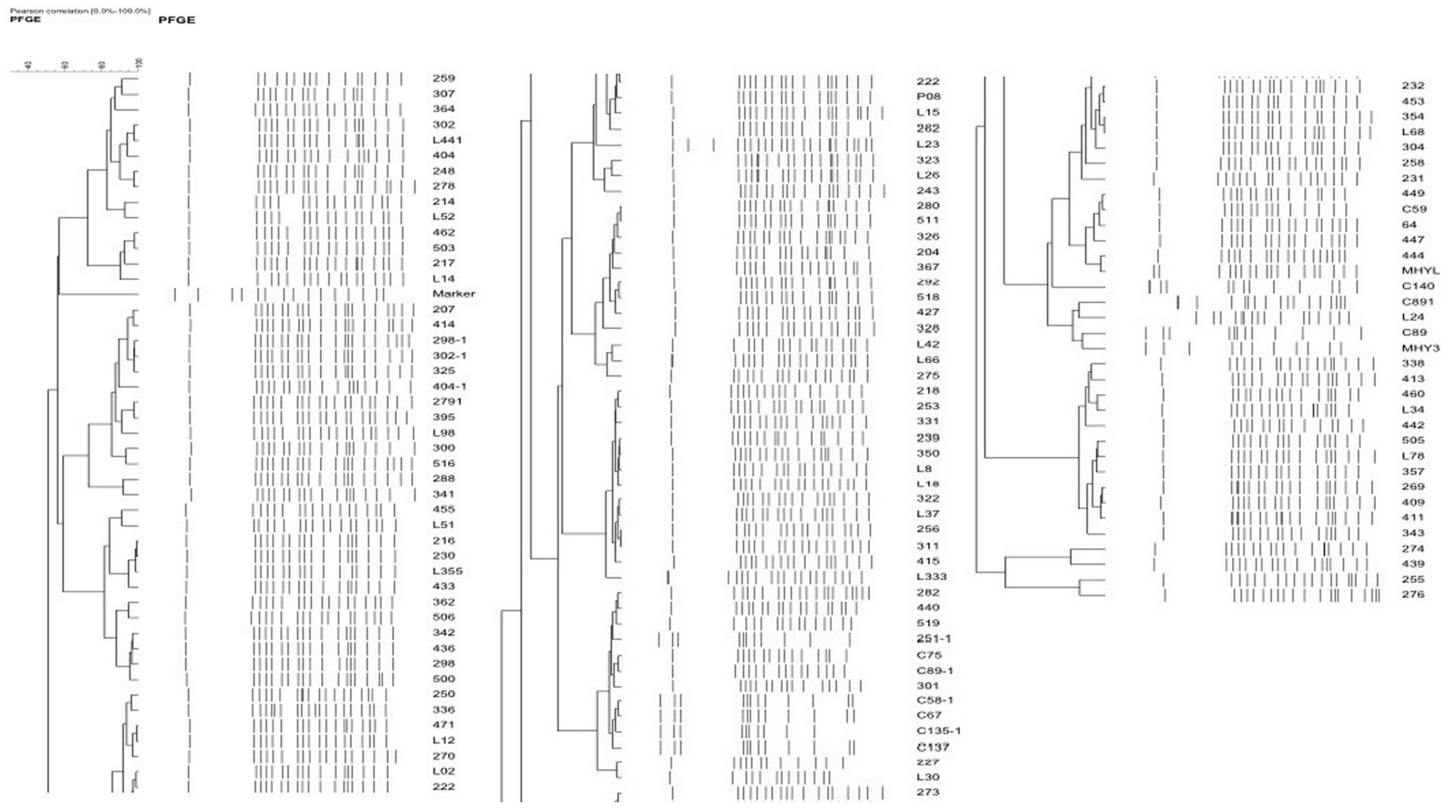
PFGE pattern of 104 Salmonella enterica serovars Indiana. Chromosomal DNA of 104 *Salmonella enterica* serovar Indiana isolates carrying the *int*1, *bla*_TEM_, *flo*R, *tet*A, *str*A, and *aac(6′)-Ib-cr* genes were digested with the restriction enzyme *Xba*I and then subjected to PFGE analysis. The results showed five major patterns as determined by PFGE.

The *int*1, *bla*_TEM_, *flo*R, *tet*A, *str*A and *aac (6') -Ib-cr* positive *Salmonella enterica* serovar Indiana isolates showed five major patterns as determined by PFGE (Figure 2). Most isolates had showed the common PFGE patterns in all 6 chicken farms, suggesting that many multidrug resistant *Salmonella enterica* serovar Indiana isolates prevailed in the three sources. Some were not derived from a specific clone, but represented a variety of different genotypes.

## Discussion

Human and animal *Salmonella* involves numerous serotypes, which exhibit certain correlation in distribution, host, separation time, and source. For example, research in Ireland from 1998 to 2003 showed that *Salmonella enterica* serovar Typhimurium was mainly isolated from cattle and swine, with the isolation rate decreasing year by year, and *Salmonella enterica* serovar Kentucky was mainly isolated from poultry [13]. A variety of serotypes were found in 137 strains of isolated food borne *Salmonella* in provinces and cities of China [14], in which the seven serotypes of *Salmonella enterica* serovar Derby, *Salmonella enterica* serovar Agona, *Salmonella enterica* serovar Enteritidis, *Salmonella enterica* serovar Reading, *Salmonella enterica* serovar Anatis, *Salmonella enterica* serovar Chester and *Salmonella enterica* serovar Typhimurium accounted for 80% of the isolates.

In this study, the multidrug resistance of 310 *Salmonella* strains to 17 antibiotics commonly used in clinical practice was examined. The investigation on drug resistance of 51 strains of human *Salmonella enterica* serovar Typhimurium conducted by Biendo [15] showed that multidrug resistance of the strains was 98%, with more than 90% of isolates resistant to sulfonamides, ampicillin, streptomycin and tetracycline, and sensitive to amikacin and cephalosporins. Analysis of the drug resistance of animal and human *Salmonella enterica* serovar Typhimurium isolated by Graziani [16] showed that 64% of strains were resistant to more than four drugs, the frequently seen drug resistance spectrum was ACSSuT type, and most strains were resistant to sulfamethoxazole. Recently, the emergence of MDR *Salmonella enterica* serovar Typhimurium, *Salmonella enterica* serovar Paratyphi and *Salmonella enterica* serovar Agona suggests that this multidrug-resistant phenotype may emerge in other *Salmonella enterica* serovar Enteritidis serotypes [17]–[18]. The existence of multidrug-resistant geneson *Salmonella enterica* serovar Indiana was not found. While the *Salmonella enterica* serovar Indiana isolated in this study was not only wide spread in the farms, but also showed characteristics of multidrug resistance. These strains had high resistance not only to streptomycin and tetracycline, but also to chloramphenicol, fluoroquinolones and cephalosporins.

Integrons are a proposed spreading mechanism of drug resistance and are widely distributed in nature [19]. Integrons have been found in human clinical isolates, avian, livestock, and animal strains, as well as in bacteria in soil and aquatic ecosystems [20]. Integrons are a movable genetic element; they can move the gene cassette by capturing and shearing mode, on the other hand, integrons were found on transposon, plasmid and other movable genetic elements, which enable their transportation and dissemination of resistance genes. Integrons can integrate drug resistance genes of almost all antimicrobial agents such as aminoglycosides, β-lactam, chloramphenicols, sulfonamides, trimethoprim, macrolides and rifampin [21]. In this study, the prevalent rate of class I integrons in *Salmonella* was 64.9% in the 310 *Salmonella* strains, which was consistent with previous findings of 59%–75% [22]. The detection rate of the class I integrons was 87.2% in the 133 *Salmonella enterica* serovar Indiana strains, and 48.3% in the 178 *Salmonella enterica* serovar Enteritidis strains, with the former slightly higher than and the latter similar to the previous report [23]. The positive rates of class I integrons in the *Salmonella enterica* serovar Indiana and *Salmonella enterica* serovar Enteritidis strains with multidrug resistance (above 5-fold resistance) were 92.5% and 92.6%, respectively, demonstrating the close relationship between multidrug resistance of strains and the prevalence of class I integrons, which was consistent with earlier research [24]–[25]. The class I integron-carrying resistance gene cassettes of *Salmonella* were mainly two gene families encoding trimethoprim and aminoglycosides (*dfr*17 and *aad*A5), as observed in other studies [26]–[27].

After the isolation of drug resistant *Salmonella* from different regions of Eastern China and analysis of the molecular epidemiology of integrons and drug resistance genes, we found that *Salmonella* was strongly resistant to antibiotics commonly used in clinical practice, and the carrying rate of integrons and drug resistance genes was positively correlated with drug resistance phenotype. This provides a scientific basis for guiding the rational clinical use of antibiotics, and for preventing and controlling the spread of drug-resistant bacteria and drug resistant genes.

PFGE analysis suggested that most pan-resistant isolates were distinctly different based on their macro-restriction patterns, which suggests that the multidrug-resistant *Salmonella* isolates carrying the *int*1, *bla*_TEM_, *flo*R, *tet*A, *str*A and *aac(6′)-Ib-cr* genes were not derived from a specific clone, but represented a wide variety of genotypes.

In conclusion, 310 *Salmonella* strains were isolated from several broiler chicken farms in Eastern China, and manifested themselves in two serotypes. A total of 282 (90.9%) *Salmonella* strains were presenting multidrug resistance. The detected carriage rate of class 1 integrons was 66.5%, with 6 strains carrying gene integron cassette *dfr*17-*aad*A5. The increasing frequency of multidrug resistance rate in *Salmonella* was associated with increasing prevalence of *int*1 genes.
